# Prognostic Effects of Inappropriate Empirical Antimicrobial Therapy in Adults With Community-Onset Bacteremia: Age Matters

**DOI:** 10.3389/fmed.2022.861032

**Published:** 2022-04-11

**Authors:** Yuan-Pin Hung, Po-Lin Chen, Ching-Yu Ho, Chih-Chia Hsieh, Chung-Hsun Lee, Ching-Chi Lee, Wen-Chien Ko

**Affiliations:** ^1^Department of Internal Medicine, Tainan Hospital, Ministry of Health and Welfare, Tainan, Taiwan; ^2^Department of Internal Medicine, National Cheng Kung University Hospital, College of Medicine, National Cheng Kung University, Tainan, Taiwan; ^3^Department of Medicine, College of Medicine, National Cheng Kung University, Tainan, Taiwan; ^4^Department of Adult Critical Care Medicine, Tainan Sin-Lau Hospital, Tainan, Taiwan; ^5^Department of Nursing, National Tainan Junior College of Nursing, Tainan, Taiwan; ^6^Department of Emergency Medicine, National Cheng Kung University Hospital, College of Medicine, National Cheng Kung University, Tainan, Taiwan; ^7^Clinical Medicine Research Center, National Cheng Kung University Hospital, College of Medicine, National Cheng Kung University, Tainan, Taiwan

**Keywords:** empirical antimicrobial, bacteremia, aging, mortality, prognosis

## Abstract

**Background:**

Studies have reported the effects of delayed administration of appropriate antimicrobial therapy (AAT) on the short-term prognosis of patients with bloodstream infections; however, whether there is an age-related difference in these effects remains debated.

**Methods:**

In this 4-year multicenter case-control study, patients with community-onset bacteremia were retrospectively categorized into the “middle-aged” (45–64 years), “old” (65–74 years), and “very old” (≥75 years) groups. Two methods were adopted to investigate the prognostic effects of delayed AAT in each age group. First, its effects were, respectively, investigated, after adjustment for the independent predictors of 30-day mortality. Second, patients in each age group were matched by the closest propensity-score (PS), which was calculated by independent predictors of mortality; the survival curves and Pearson chi-square tests were adopted to disclose its effects in each PS-matching group.

**Results:**

Each hour of delayed AAT resulted in an average increase in the 30-day crude mortality rate of 0.2% (*P* = 0.03), 0.4% (*P* < 0.001), and 0.7% (*P* < 0.001) in middle-aged (968 patients), old (683), and very old (1,265) patients, after, respectively, adjusting the independent predictors of mortality in each group. After appropriate PS-matching, no significant proportion differences in patient demographics, bacteremia characteristics, severity of bacteremia and comorbidities, and 15-day or 30-day crude mortality rates were observed between three matched groups (582 patients in each group). However, significant differences in survival curves between patients with delayed AAT > 24 or >48 h and those without delayed administration were demonstrated in each age group. Furthermore, the odds ratios of 30-day mortality for delayed AAT > 24 or >48 h were 1.73 (*P* = 0.04) or 1.82 (*P* = 0.04), 1.84 (*P* = 0.03) or 1.95 (*P* = 0.02), and 1.87 (*P* = 0.02) or 2.34 (*P* = 0.003) in the middle-aged, old, and very old groups, respectively. Notably, the greatest prognostic impact of delayed AAT > 24 or >48 h in the very old group and the smallest impact in the middle-aged group were exhibited.

**Conclusion:**

For adults (aged ≥45 years) with community-onset bacteremia, the delayed AAT significantly impacts their short-term survival in varied age groups and the age-related differences in its prognostic impact might be evident.

## Introduction

Community-onset bacteremia is a common problem encountered by physicians, because of its annual incidence of up to 0.15% in the community and the case-fatality rate of up to 17% (1). The hemodynamic support and prompt administration of appropriate antimicrobial therapy (AAT) can result in survival benefits for patients with bacteremia (2–5), particularly those initially experienced the critical illness (2, 5). However, concerning the increased proportion of causative microorganisms harboring antibiotic-resistant genes worldwide (6), prompt administration of AAT remains challenging for first-line clinicians.

Appropriate management has always been crucial but difficult for older patients with bloodstream infections, because the bacteremia incidence and mortality rate markedly increase with age (7) and older patients also experience the non-specific and atypical presentation at bacteremia onset (8). Moreover, a recent study reported that a positive age-related trend in bacteremia severity (9). Of importance, the higher incidence of antimicrobial-resistant causative microorganisms resulted in the more prolonged AAT delay in older patients (9). However, information detailing the influence of the aging immunity on the effectiveness of antimicrobial therapy is limiting. Therefore, we hypothesized that the prognostic impacts of delayed AAT would be enhanced with age, because of the aging of the immunity response to systematic infections. In the present study, we aimed to investigate the differential effects of delayed AAT on the short-term prognoses of adults with community-onset bacteremia among the varied age groups.

## Materials and Methods

### Study Design and Sites

Between January 2015 and December 2018, we retrospectively conducted a 4-year multicenter case-control study at the emergency departments (EDs) of three hospitals in southern Taiwan, namely a university-affiliated medical center (1,300 beds) and two teaching hospitals (one with 460 beds and one with 380 beds). The aimed patient was a naïve adult (i.e., without any antimicrobial and medical therapy prior to ED arrival) with community-onset bacteremia in the ED. According to the cut-off age suggested in the previously established study (10), the eligible patients were grouped as the following: middle aged (45–64 years), old (65–74 years), and very old (≥75 years). This study was approved by the Institutional Review Board of study hospitals, and the requirement of obtaining informed consent was waived. Clinical information was obtained and reported according to the Strengthening the Reporting of Observational Studies in Epidemiology (11).

### Patient Selection

From a database search, we identified adult patients who had undergone blood culture sampling at the ED. Among patients who had multiple episodes of bloodstream infections during the study period, we examined only the first episode. Of patients aged ≥45 years and experienced the bacterial growth of blood cultures, we excluded those with contaminated blood cultures, mycobacteremia, hospital-onset bacteremia, bacteremia diagnosed or treated before ED arrival, an uncertain mortality date, and incomplete chart records. Consequently, the study consisted of naïve patients aged ≥45 years experienced community-onset bacteremia was established.

### Data Collection

Using a predetermined form, clinical data were jointly collected by a board-certified ED physician and an infectious disease (ID) physician who were well-trained by the IRB course and blinded to the aim and hypothesis of this study. Any discrepancy in data capturing was resolved through the discussion between two abstractors in periodic meetings. The captured ED information included patient demographics, vital signs, laboratory data, comorbidity types and severity, severity of bacteremia [Pitt bacteremia score (PBS)], timing and types of antimicrobial administration, and the length of stay. Further data regarding the length of hospitalization, durations and types of antimicrobial agents administered, imaging findings, timing and types of surgical and radiological interventions, causative microorganisms, bacteremia sources, and 30-day mortality were collected. The primary study endpoint was crude 30-day mortality after ED arrival (i.e., bacteremia onset).

### Microbiological Methods

Blood cultures were incubated in a Bactec 9240 instrument (Becton Dickinson Diagnostic Systems, Sparks, MD, United States). The causative microorganism was prospectively stored after identification by the Gram-Negative-Identification Card of the Vitek system (bioMérieux, Lyon, France) or the matrix-assisted laser desorption ionization time-of-flight mass spectrometry (MALDI-TOF MS) and susceptibility testing, which was performed using the disk diffusion method for aerobes and the agar dilution method for anaerobes, in accordance with the contemporary guideline of the Clinical and Laboratory Standards Institute (CLSI) (12). The susceptibility of Gram-negative aerobes to the following was tested: ampicillin/sulbactam, piperacillin/tazobactam, levofloxacin, moxifloxacin, cefazolin, cefuroxime, cefotaxime, ceftazidime, cefepime, ertapenem, and imipenem. For streptococci, staphylococci, and enterococci, the tested antibiotics were penicillin, cefoxitin, and ampicillin, respectively. Furthermore, the susceptibility of anaerobes to ampicillin/sulbactam, piperacillin/tazobactam, metronidazole, and moxifloxacin was tested. To determine the AAT timing for each patient, provided that empirical antibiotics administered were not included in the initial susceptibility panel, the susceptibility to the indicated antibiotic was tested.

### Definitions

Community-onset bacteremia refers to the bacteremic onset within the community (1, 3). Contaminated blood cultures were defined as those exhibiting the growth of potential contaminating pathogens, such as Gram-positive bacilli, *Micrococcus* species, *Bacillus* species, and *Propionibacterium* species (13). According to the previous suggestion (3), antimicrobial therapy was appropriate when the following criteria were totally fulfilled: (i) all bacteremia-causing pathogens in one episode were *in vitro* susceptible to the antimicrobials administered according to the contemporary CLSI criteria (12) and (ii) the route and dosage of antimicrobials was administered as recommended in the Sanford Guide 2021 (14). The period of AAT delays was defined as the gap between the onset of bacteremia (ED arrival) and the first dose of AAT administration (3).

The severity of bacteremia was determined according to the PBS, which was calculated during the first 24 h after ED arrival, and patients with a PBS of ≥4 were categorized as critically ill (3). Comorbidities were defined as described previously (15). Preexisting neurological diseases majorly included the Alzheimer’s disease; diseases resulting in dementia, multiple sclerosis, and demyelinating diseases; and stroke and cerebrovascular events (16). Hemato-oncological comorbidities consisted of hematological malignancies and solid tumors under active treatment. The severity of comorbidities was assessed based on the classification of McCabe and Jackson (rapidly fatal, ultimately fatal, and non-fatal) (17). Crude mortality was defined as death from all causes.

Consistent with general concepts (18), a patient was assigned to one of the bacteremia sources based on clinical diagnoses and/or isolation of pathogens. Based on the recommendation of the Surviving Sepsis Campaign (SSC) (13), complicated bacteremia was equated with whether that the bacteremia source is amenable to undergo source control, and the adequateness of source control for complicated bacteremia was determined by the board-certified ID physicians.

### Statistical Analyses

Statistical analyses were performed using the Statistical Package for Social Science for Windows (version 23.0; Chicago, IL, United States). Categorical and continuous clinical variables were compared using the Pearson chi-square test or the Fisher’s exact test, if the expected value was <5, and the independent *t* test, respectively. Spearman’s correlation was calculated to analyze the linear-by-linear association between the period of delayed ATT and mortality rates. A *E*-value was calculated to examine the potential effect of unmeasured confounders in our study (19). A two-sided *P* value of <0.05 was considered significant.

Two methods were adopted to investigate the prognostic effects of delayed AAT in the different age groups. First, the significant predictors of 30-day mortality were recognized by the univariable analysis. Consistent with previous methods (3, 20), the significant prognostic predictors and the continuous covariable of “the hour of delayed AAT” were jointly processed under the model of backward and stepwise logistic regression in each age group. Second, similar to the previous study (4), we performed the propensity-score (PS)-matched analysis to overcome baseline differences between three age groups. The PS was calculated by the independent determinants of 30-day crude mortality in overall patients, recognized by the model of backward and stepwise logistic regression. Patients in the different age groups were manually matched at a ratio of 1:1:1, by the closest total propensity-scores. Like the previous rule (21), the matching tolerance for the PS difference was *P* < 0.2. Focusing on each PS-matched group, the Kaplan–Meier curve and log rank test were utilized for the 30-day survival analysis, and the Pearson chi-square test were used to assess the prognostic effect of delayed AAT.

## Results

### Demographic Characteristics of the Entire Patients

Of 5,786 patients with bacterial growth in blood cultures, 2,916 were eligible as the aimed population based on the inclusion and exclusion criteria; and 968 (33.2%), 683 (23.4%), and 1,265 (43.4%) were categorized in the middle-aged, old, and very old groups, respectively (Supplementary Figure 1). Of the total 2,916 patients, the majority (2,370, 81.3%) were admitted to general wards through the ED, 304 (10.4%) were admitted to intensive care units, 100 (3.4%) died in the ED, and only 142 (4.9%) were discharged through the ED and subsequently followed at outpatient clinics. The 609 (21.2%) patients were critically ill (i.e., PBS ≥ 4) upon ED arrival, and 15- and 30-day crude mortality rates were 12.0% (349 patients) and 15.4% (449), respectively.

### Clinical Manifestations and Clinical Outcomes in the Different Age Groups

Table 1 presents a comparison of clinical manifestations and outcomes between the middle-aged, old, and very old groups. Significant differences were noted in the proportion of male patients, nursing-home residents, comorbid severity (McCabe and Jackson classification), major comorbidities, severe bacteremia episodes (PBS ≥ 4 at onset), specific bacteremia sources (i.e., pneumonia, skin and soft-tissue infections, primary bacteremia, biliary tract infections, and liver abscess), and the presence of polymicrobial bacteremia among the three groups. Despite the similar periods of delayed AAT, intravenous (IV) antimicrobial therapy, and hospitalization as well as similar 15-day or 30-day mortality rates in the varied age groups, the proportions of approximately half of empirical antimicrobial classes differed in the varied age groups (Supplementary Table 1).

**TABLE 1:** Clinical characteristics and outcomes of the overall and propensity-score-matched adults in varied age groups*.

Clinical variable	Overall patients				Matched patients			
	Patient number (%)			*P* value	Patient number (%)			*P* value
	Middle-aged *n* = 968	Old *n* = 683	Very old *n* = 1265		Middle-aged *n* = 582	Old *n* = 582	Very old *n* = 582	
Gender, male	**534 (55.2)**	**309 (45.2)**	**644 (50.9)**	** < 0.001**	299 (51.4)	260 (44.7)	287 (49.3)	0.06
Nursing-home residence	**27 (2.8)**	**31 (4.5)**	**115 (9.1)**	** < 0.001**	16 (2.7)	22 (3.8)	20 (3.4)	0.61
Fatal comorbidity (McCabe and Jackson classification)	**285 (29.4)**	**195 (28.6)**	**264 (20.9)**	** < 0.001**	161 (27.7)	163 (28.0)	141 (24.2)	0.27
**Major comorbidity**								
Cardiovascular disease	**367 (37.9)**	**406 (59.4)**	**907 (71.7)**	** < 0.001**	**219 (37.6)**	**347 (59.6)**	**402 (69.1)**	** < 0.001**
Diabetes mellitus	**365 (37.7)**	**320 (46.9)**	**483 (38.2)**	** < 0.001**	230 (39.5)	263 (45.2)	231 (39.7)	0.08
Hemato-oncology	**308 (31.8)**	**239 (35.0)**	**351 (27.7)**	**0.003**	196 (33.7)	196 (33.7)	195 (33.5)	0.98
Chronic kidney disease	**144 (14.9)**	**131 (19.2)**	**276 (21.8)**	** < 0.001**	94 (16.2)	115 (19.8)	121 (20.8)	0.11
Liver cirrhosis	**170 (17.6)**	**95 (13.9)**	**100 (7.9)**	** < 0.001**	65 (11.2)	65 (11.2)	64 (11.0)	0.99
Neurological disease	87 (9.0)	140 (20.5)	497 (39.3)	< 0.001	60 (10.3)	80 (13.7)	77 (13.2)	0.16
**Major source of bacteremia**								
Urinary tract	291 (30.1)	240 (35.1)	431 (34.1)	0.05	205 (35.2)	205 (35.2)	200 (34.4)	0.94
Intra-abdominal	130 (13.4)	78 (11.4)	133 (10.5)	0.10	65 (11.2)	68 (11.7)	83 (14.3)	0.23
Pneumonia	**115 (11.9)**	**88 (12.9)**	**244 (19.3)**	** < 0.001**	74 (12.7)	73 (12.5)	77 (13.2)	0.94
Skin and soft-tissue	**111 (11.5)**	**52 (7.6)**	**108 (8.5)**	**0.01**	61 (10.5)	47 (8.1)	51 (8.8)	0.34
Primary bacteremia	**80 (8.3)**	**56 (8.2)**	**72 (5.7)**	**0.03**	43 (7.4)	48 (8.2)	44 (7.6)	0.85
Biliary tract	**59 (6.1)**	**63 (9.2)**	**140 (11.1)**	** < 0.001**	46 (7.9)	52 (8.9)	55 (9.5)	0.64
Liver abscess	**43 (4.4)**	**24 (3.5)**	**31 (2.5)**	**0.03**	16 (2.7)	16 (2.7)	17 (2.9)	0.98
Polymicrobial bacteremia	**74 (7.6)**	**64 (9.4)**	**151 (11.9)**	**0.003**	51 (8.8)	51 (8.8)	50 (8.6)	0.99
Inadequate source control during antibiotic therapy	34 (3.5)	20 (2.9)	39 (3.1)	0.77	12 (2.1)	13 (2.2)	14 (2.4)	0.92
Pitt bacteremia score ≥4 at onset	**168 (17.4)**	**144 (21.1)**	**297 (23.5)**	**0.005**	97 (16.7)	111 (19.1)	109 (18.7)	0.52
**Duration, median (interquartile range)**								
Delayed AAT, hour	2 (1–7)	2 (1–7)	2 (1–10)	0.82	2 (1–8)	2 (1–8)	2 (1–6)	0.88
IV antimicrobial therapy, day	8 (5–15)	5 (5–15)	8.5 (5–15)	0.11	8 (4–14)	5 (8–15)	8 (5–15)	0.56
Length of hospitalization, day	10 (6–17)	10 (6–18)	10 (6–17)	0.13	10 (5–18)	10 (6–18)	10 (6–17)	0.87
**Crude mortality rate**								
15-day	111 (11.5)	75 (11.0)	163 (12.9)	0.39	72 (12.4)	59 (10.1)	70 (12.0)	0.44
30-day	138 (14.3)	104 (15.2)	207 (16.4)	0.39	87 (14.9)	82 (14.1)	87 (14.9)	0.89

AAT, appropriate antimicrobial therapy; IV, intravenous. *Data are expressed as numbers (%), unless indicated specifically. Boldface indicates statistical significance (P < 0.05) between groups in the univariable analysis.

### Distributions of Causative Microorganisms in the Different Age Groups

Of the total 3,281 causative microorganisms (middle-aged, old, and very old: 1,065, 762, and 1,454 isolates, respectively), the leading were *Escherichia coli* (1,222, 37.2%), *Klebsiella pneumoniae* (455, 13.9%), *Staphylococcus aureus* (375, 11.4%), *Streptococcus* species (355, 10.8%), *Pseudomonas aeruginosa* (108, 3.3%), *Enterococcus* species (106, 3.2%), *Enterobacter cloacae* (89, 2.7%), *Proteus mirabilis* (76, 2.3%), *Salmonella enteritidis* (48, 1.5%), and *Aeromonas* species (45, 1.4%). Notably, dissimilar specie distributions among the different age groups were observed in five common microorganisms, in terms of *E. coli*, *K. pneumoniae*, *S. aureus*, *S. pneumoniae*, and *Enterococcus faecalis* (Supplementary Table 2).

Overall, methicillin-resistant *S. aureus*, ampicillin-susceptible enterococci, and penicillin-susceptible streptococci accounted for 39.4% (148 isolates) of *S. aureus*, 85.8% (91) of enterococci, and 93.5% (332) of streptococci, respectively. Meanwhile, cefazolin, cefuroxime, ampicillin/sulbactam, moxifloxacin, cefotaxime, levofloxacin, ceftazidime, ertapenem, cefepime, piperacillin/tazobactam, and imipenem were active against 50.6, 71.2, 74.3, 81.3, 78.1, 82.2, 83.6, 88.9, 93.0, 94.6, and 99.3% of Gram-negative aerobes, respectively. Ampicillin/sulbactam, moxifloxacin, piperacillin/tazobactam, and metronidazole were active against 78.0, 87.5, 94.0, and 97.6% of the total anaerobes, respectively.

### Predictors of 30-Day Mortality in Overall Patients

Of numerous predictors of 30-day mortality recognized by the univariable analysis (Supplementary Table 3), the following was independently associated with 30-day crude mortality using the multivariable regression model: nursing-home residence; polymicrobial bacteremia; critically ill (PBS ≥ 4) patients at onset; inadequate source control; bacteremic pneumonia; bacteremia due to urinary tract infections or liver abscess; fatal comorbidities (McCabe and Jackson classification); and comorbidities of hemato-oncology or liver cirrhosis. Notably, the hourly AAT delay was associated with an average increase of 0.3% in 30-day crude mortality rates [adjusted odds ratio (AOR), 1.003; *P* < 0.001].

### Prognostic Effects of Delayed Appropriate Antimicrobial Therapy in the Different Age Groups

**FIGURE 1:**
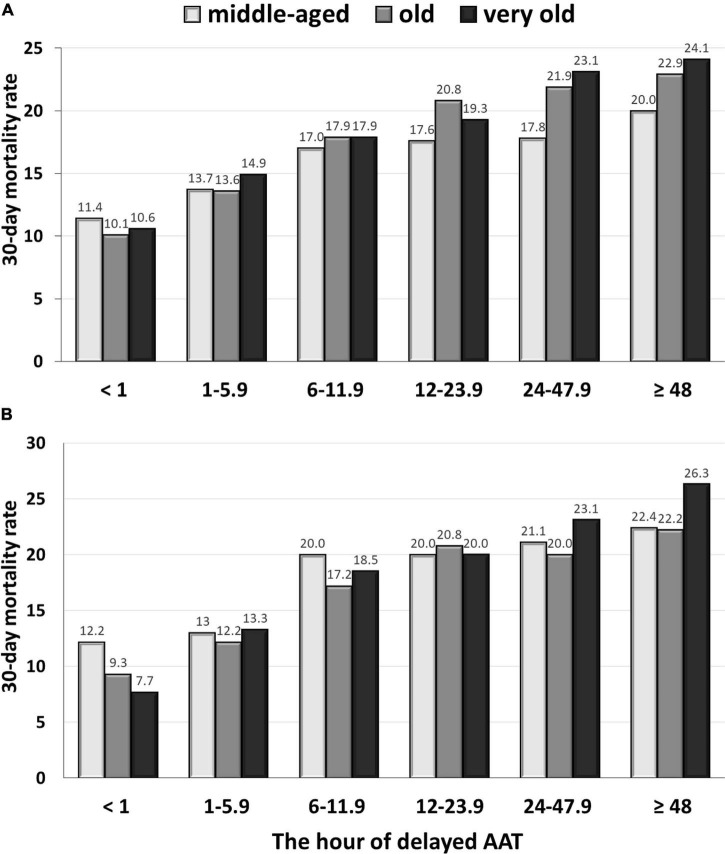
The delayed AAT-related trend in 30-day crude mortality rates among the middle-aged, old, and very old groups: the overall **(A)** and propensity score-matched **(B)** patients. AAT, appropriate antimicrobial therapy.

A positive delayed AAT-related trend in 30-day mortality rates among the middle-aged (γ = 0.995, *P* < 0.001), old (γ = 0.969, *P* = 0.001), and very old (γ = 0.988, *P* < 0.001) groups was disclosed (Figure 1A). Among 968 patients in the middle-aged group (Table 2), after adjustment for the independent predictors of 30-day mortality determined in the multivariable analysis [i.e., the critical illness at onset, bacteremic pneumonia, and fatal comorbidities (McCabe and Jackson classification)], the prognostic impact of the hourly AAT delay was significant (AOR, 1.002; *P* = 0.03). Moreover (Table 2), the prognostic effect of 1-h AAT delay remained independently significant in the old age (AOR, 1.004; *P* < 0.001) and very old (AOR, 1.007; *P* < 0.001) groups, after respective adjustment for the independent predictors of 30-day mortality.

**TABLE 2:** Prognostic impacts of hourly AAT delays in the middle-aged, old, and very old groups.

Variable	Patient number (%)*		Univariable analysis		Multivariable analysis	
	Death	Survival	OR (95% CI)	*P* value	Adjusted OR (95% CI)	*P* value
**The middle-aged patient (*n* = 968)**	*n* = 138	*n* = 830				
Delayed AAT, hour, median (IQR)	2.0 (1.0–21.2)	2.0 (0.9–6.0)	–	<0.001	**1.002 (1.000–1.004)**	**0.03**
Gender, male	96 (69.6)	438 (52.8)	2.05 (1.39–3.01)	< 0.001	NS	NS
Nursing-home residence	10 (7.2)	17 (2.0)	3.74 (1.67–8.34)	0.001	NS	NS
Polymicrobial bacteremia	23 (16.7)	51 (6.1)	3.06 (1.80–5.19)	< 0.001	NS	NS
Pitt bacteremia score ≥4	85 (61.6)	83 (10.0)	14.43 (9.57–21.78)	<0.001	**12.39 (7.75–19.81)**	** < 0.001**
**Source of bacteremia**						
Pneumonia	52 (37.7)	63 (7.6)	7.36 (4.79–11.31)	<0.001	**4.11 (2.37–7.12)**	** < 0.001**
Urinary tract infection	16 (11.6)	275 (33.1)	0.27 (0.15–0.46)	< 0.001	0.57 (0.30–1.08)	0.09
Liver abscess	1 (0.7)	42 (5.1)	0.14 (0.02–1.00)	0.02	0.14 (0.02–1.14)	0.07
Fatal comorbidity (McCabe and Jackson classification)	80 (58.0)	205 (24.7)	4.21 (2.90–6.11)	<0.001	**2.26 (1.33–3.83)**	**0.002**
**Comorbidity**						
Hemato-oncology	80 (58.0)	228 (27.5)	3.64 (2.51–5.28)	< 0.001	1.73 (1.00–3.00)	0.05
Diabetes mellitus	40 (29.0)	325 (39.2)	0.63 (0.43–0.94)	0.02	NS	NS
Liver cirrhosis	36 (26.1)	134 (16.1)	1.83 (1.20–2.80)	0.004	NS	NS
**The old patient (*n* = 683)**	*n* = 104	*n* = 579				
Delayed AAT, hour, median (IQR)	3.0 (2.0–39.3)	2.0 (1.0–6.0)	–	<0.001	**1.004 (1.002–1.006)**	** < 0.001**
Gender, male	61 (58.7)	248 (42.8)	1.89 (1.24–2.89)	0.003	NS	NS
Pitt bacteremia score ≥4	65 (62.5)	79 (13.6)	10.55 (6.64–16.75)	<0.001	**11.08 (6.40–19.19)**	** < 0.001**
**Source of bacteremia**						
Pneumonia	35 (33.7)	53 (9.2)	5.03 (3.07–8.26)	< 0.001	1.88 (1.00–3.53)	0.05
Urinary tract infection	16 (15.4)	224 (38.7)	0.29 (0.17–0.50)	<0.001	**0.50 (0.26–0.97)**	**0.04**
Fatal comorbidity (McCabe and Jackson classification)	56 (53.8)	139 (24.0)	3.69 (2.40–5.68)	<0.001	**4.90 (2.88–8.33)**	** < 0.001**
Comorbid hemato-oncology	55 (52.9)	184 (31.8)	2.41 (1.58–3.68)	< 0.001	NS	NS
**The very old patient (*n* = 1265)**	*n* = 207	*n* = 1058				
Delayed AAT, hour, median (IQR)	3.0 (1.2–40.0)	2.0 (1.1–8.0)	–	<0.001	**1.007 (1.004–1.011)**	** < 0.001**
Nursing-home Residence	35 (16.9)	80 (7.6)	2.49 (1.62–3.82)	<0.001	**1.81 (1.04–3.15)**	**0.03**
Polymicrobial bacteremia	41 (19.8)	110 (10.4)	2.13 (1.43–3.16)	<0.001	**1.71 (1.03–2.82)**	**0.04**
Pitt bacteremia score ≥4	140 (67.6)	157 (14.8)	1.88 (1.37–2.56)	<0.001	**9.27 (6.32–15.60)**	** < 0.001**
Inadequate source control during antibiotic therapy	18 (8.7)	21 (2.0)	4.70 (2.50–8.99)	<0.001	**4.70 (2.10–10.54)**	** < 0.001**
**Source of bacteremia**						
Pneumonia	86 (41.5)	158 (14.9)	0.05 (2.93–5.60)	< 0.001		
Urinary tract infection	18 (8.7)	413 (39.0)	0.15 (0.09–0.25)	<0.001	**0.19 (0.11–0.33)**	** < 0.001**
Biliary tract infection	8 (3.9)	132 (12.5)	0.28 (0.14–0.59)	<0.001	**0.21 (0.09–0.50)**	** < 0.001**
Fatal comorbidity (McCabe and Jackson classification)	78 (29.5)	129 (12.9)	2.84 (2.05–3.91)	<0.001	**2.66 (1.77–4.01)**	** < 0.001**
**Comorbidity**						
Hypertension	121 (58.5)	695 (65.7)	0.74 (0.54–1.00)	0.047	NS	NS
Hemato-oncology	81 (39.1)	270 (25.5)	1.88 (1.37–2.56)	<0.001	NS	NS
Liver cirrhosis	31 (15.0)	69 (6.5)	2.53 (1.61–3.97)	<0.001	**2.60 (1.46–4.64)**	**0.001**
Urological disease	16 (7.7)	137 (12.9)	0.56 (0.33–0.97)	0.04	NS	NS

AAT, appropriate antimicrobial therapy; CI, confidence interval; IQR, interquartile range; OR, odds ratio; NS, not significant (by backward multivariable regression). Boldface indicates statistical significance (P < 0.05) in the logistic regression model. *Data are expressed as numbers (%), unless indicated specifically.

### Prognostic Impacts of Delayed Appropriate Antimicrobial Therapy on Propensity-Score-Matched Patients in the Different Age Groups

Based on the closest propensity scores using ten independent determinants of mortality (as shown in Supplementary Table 3), 582 patients in each age group were matched. After adequate PS-matching (Table 1), no significant differences of the patient proportion were exhibited between three age groups, in terms of patient characteristics, the proportion of nursing-home residents, fatal comorbidities (McCabe and Jackson classification), major comorbidities, major sources of bacteremia, polymicrobial bacteremia, inadequate source control, or severe bacteremia (PBS ≥ 4) at onset, the period of delayed AAT, IV antimicrobial therapy, or hospitalization, and 15-day or 30-day crude mortality rates. Of note, the dissimilar proportion between three age groups was only observed in comorbid cardiovascular diseases.

**FIGURE 2:**
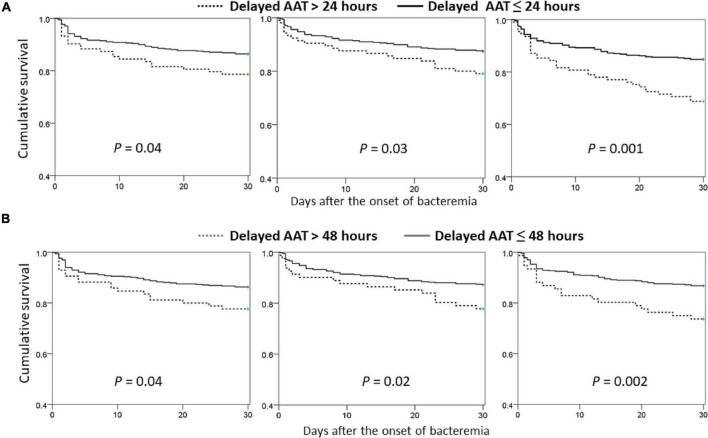
Comparisons of Kaplan–Meier survival curves between the propensity-score-matched patients with delayed AAT > 24 **(A)** or >48 **(B)** hours and those without delayed administration, categorized by the different age groups. AAT, appropriate antimicrobial therapy.

Focusing on the matched groups, the positive delayed-AAT-related trends in 30-day mortality rate among the middle-aged (γ = 0.921, *P* = 0.009), old (γ = 0.949, *P* = 0.004), and very old (γ = 0.982, *P* < 0.001) groups were disclosed (Figure 1B). Notably, the highest and lowest coefficient of correction in the very old and middle-aged groups were disclosed, respectively. Further analyses in survival curves, a significant difference between treatment with delayed AAT > 24 h (Figure 2A) or >48 h (Figure 2B) and treatment without delay was consistently demonstrated in three age groups. Of importance, the largest difference in survival curves in the very old group and the smallest difference in the middle-aged group occurred, in responses to inadequate empirical antibiotic therapy (i.e., delayed AAT > 24 or >48 h).

In further analyses on the matched groups (Table 3), the prognostic impacts of delayed AAT > 24 h remained significant in the middle-aged [odds ratio (OR), 1.73; *P* = 0.04], old (OR, 1.84; *P* = 0.03), and very old (OR, 1.87, *P* = 0.02) groups. Similarly, the prognostic impacts of delayed AAT > 48 h remained significance in the middle-aged (OR, 1.82; *P* = 0.04), old (OR, 1.95; *P* = 0.02), and very old (OR, 2.34, *P* = 0.003) groups. In sum, regardless of whether delayed AAT > 24 or 48 h, the vast prognostic impacts (i.e., the largest OR) of inadequate empirical antibiotic therapy occurred in the very old group.

**TABLE 3:** Prognostic effects of delayed AAT in the propensity score-matched patients, categorized by the different age groups.

Variable	30-day mortality rate (%)	Odds ratio (95% CI)	*P* value
**Middle-aged (*n* = 582)**			
Delayed AAT **≥** 24 h (*n* = 103)	21.4	1.73 (1.01–2.96)	0.04
Delayed AAT **≥** 48 h (*n* = 85)	22.4	1.82 (1.03–3.21)	0.04
**Old (*n* = 582)**			
Delayed AAT **≥** 24 h (*n* = 104)	20.2	1.84 (1.07–3.17)	0.03
Delayed AAT **≥** 48 h (*n* = 81)	22.2	1.95 (1.09–3.51)	0.02
**Very old (*n* = 582)**			
Delayed AAT **≥** 24 h (*n* = 98)	22.4	1.87 (1.09–3.21)	0.02
Delayed AAT **≥** 48 h (*n* = 76)	26.3	2.34 (1.32–4.15)	0.003

*CI, confidence interval.*

## Discussion

Although some studies have reported that the prompt AAT treatment has no effect in reducing patient fatality (5, 22), numerous bacteremia studies have consistently reported the significant impact of delayed AAT on short-term mortality of patient populations with vast heterogeneity, such as the elderly (23), critically ill individuals (2, 5), neutropenia (24), and those infected by specific pathogens [i.e., *P. aeruginosa* (25), *S. aureus* (26), and Enterobacteriaceae (27)]. Consistent with previous studies regarding community-onset/acquired bacteremia (2–4), we observed that the delayed AAT administration significantly impacts on the survival of bacteremic patients in each age group. More importantly, in conformity with our hypothesis that the aging immunity could affect the effectiveness of antimicrobial therapy, our study revealed that the adverse effects of delayed AAT administration on short-term mortality increased with patients’ age.

Compared to the middle-aged adults, more prognostic advantage of prompt AAT administration in the older individual experiencing bacteremia has been emphasized in our study. We believed that the aging of the immune system resulted in the age-related difference in its prognostic effect, as demonstrated herein. Like a double-edged sword, a diminished response caused by the immunity aging leads to increased rates of infections, and on the other hand, the altered immunity response to infections, resulting in atypical presentations and thereby delayed diagnosis and treatment, might couple with more severe infections (7). Therefore, concerning the prognostic disadvantage of delayed AAT in bacteremia episodes, adopting a useful predictor or early detector for the elderly to avoid delayed treatment is recommended. Moreover, because of atypical presentations, early diagnosing and managing of bacteremia in the older population is challenging (7, 8). To achieve appropriate administration of empirical antimicrobials in the elderly, the prompt administration of broad-spectrum antimicrobials for those with suspected bloodstream infections and lowering the threshold of prescribing antibiotics for the first-line clinician are the essential.

Consistent with previous reports (7, 9), the dissimilarity in bacteremia severity and the distribution of bacteremia sources and causative microorganism in different patent’s age was observed in present study. We concerned that faster AAT administration is essential in improving the prognoses of older patients by a corresponding increase in their bacteremia severity and the baseline characteristics at bacteremia onset greatly varied in three age groups. Accordingly, in addition to use the model of multivariate regression to overcome the confounding factors inside each age group, we use the PS-matching to reduce the dissimilarity of baseline characteristics between three age groups. Despite a significant difference in comorbid cardiovascular diseases between the age groups, we believed that the appropriate matching had been achieved herein because of the limited association of cardiovascular diseases and the prognoses of bacteremic patients in the literature. Consequently, by two different statistical methods, our study consistently indicated the dissimilar impacts of delayed ATT on short-term prognoses of varied age patients.

This study has several limitations. First, the retrospective nature of this study made it prone to recall bias during data collection. To reduce this bias, all clinical information was retrieved by two physicians blinded to the study aims and in a double-checking manner. Second, to investigate the prognostic effects of delayed AAT, patients with incomplete clinical information or uncertain date of death were excluded. Selection bias may be trivial as a result of the few proportion of excluded patients in this study. Third, because previous studies have reported the neglected difference in therapeutic efficacies between narrow- and broad-spectrum antimicrobials administered as appropriate empirical agents (28, 29), the differential efficacy between various types of empirical antimicrobials was not considered in our analyses, as the design in previously reported studies (2–5, 23–27). Superior to these studies, the present study designed to further collect more prognostic determinants, such as source control for complicated bacteremia, and a *E*-value was calculated for the existence of confounding factors affecting our results. Because of extreme low *E*-values (i.e., 1.05 in overall patients), unmeasured confounders in our study should be trivial. Finally, because study hospitals were located in southern Taiwan, the results of this study should be validated in other communities.

Conclusively, for adults aged ≥45 years and experiencing community-onset bacteremia, the delayed AAT significantly impacts their survival. Notably, age-related differences in the adverse effects of delayed AAT administration on short-term prognoses might be evident. Therefore, to facilitate the prompt AAT administration, epidemiological surveillance, rapid pathogen and susceptibility identification, and the incorporation of broad-spectrum antimicrobials as the empirical agents into the antibiotic stewardship program is warranted, particularly in the older patients.

## Data Availability Statement

The original contributions presented in the study are included in the article/Supplementary Material, further inquiries can be directed to the corresponding authors.

## Ethics Statement

The studies involving human participants were reviewed and approved by the Institutional Review Board of National Cheng Kung University Hospital (B-ER-109-114), Madou Sin-Lau Hospital (SLH 9919-108-006), and Tainan Sin-Lau Hospital (SLH 9919-108-009). Written informed consent for participation was not required for this study in accordance with the national legislation and the institutional requirements.

## Author Contributions

C-CL and W-CK conceived the study idea and designed the study. Y-PH, P-LC, C-YH, C-CH, and C-HL provided the data collection and chart reviews. C-CL, Y-PH, and P-LC provided the data of microbiological analyses. C-CL and C-YH provided methodological and statistical advice on the study design and data analysis. Y-PH, C-CL, and W-CK provided expertise in infectious disease. C-CL drafted the manuscript. W-CK revised the manuscript carefully from a professional point of view. All authors read and approved the final manuscript.

## Conflict of Interest

The authors declare that the research was conducted in the absence of any commercial or financial relationships that could be construed as a potential conflict of interest.

## Publisher’s Note

All claims expressed in this article are solely those of the authors and do not necessarily represent those of their affiliated organizations, or those of the publisher, the editors and the reviewers. Any product that may be evaluated in this article, or claim that may be made by its manufacturer, is not guaranteed or endorsed by the publisher.
