# Supplementary material for: Prognostic Effects of Inappropriate Empirical Antimicrobial Therapy in Adults With Community-Onset Bacteremia: Age Matters
Source: Front Med (Lausanne). 2022 Apr 11;9:861032. doi: 10.3389/fmed.2022.861032 (PMC9037591; doi:10.3389/fmed.2022.861032)
Supplement: Supplementary file 1 [file Data_Sheet_1.pdf]

SUPPLEMENTAL DATA

Supplemental Figure 1. Flowchart of patient selections.

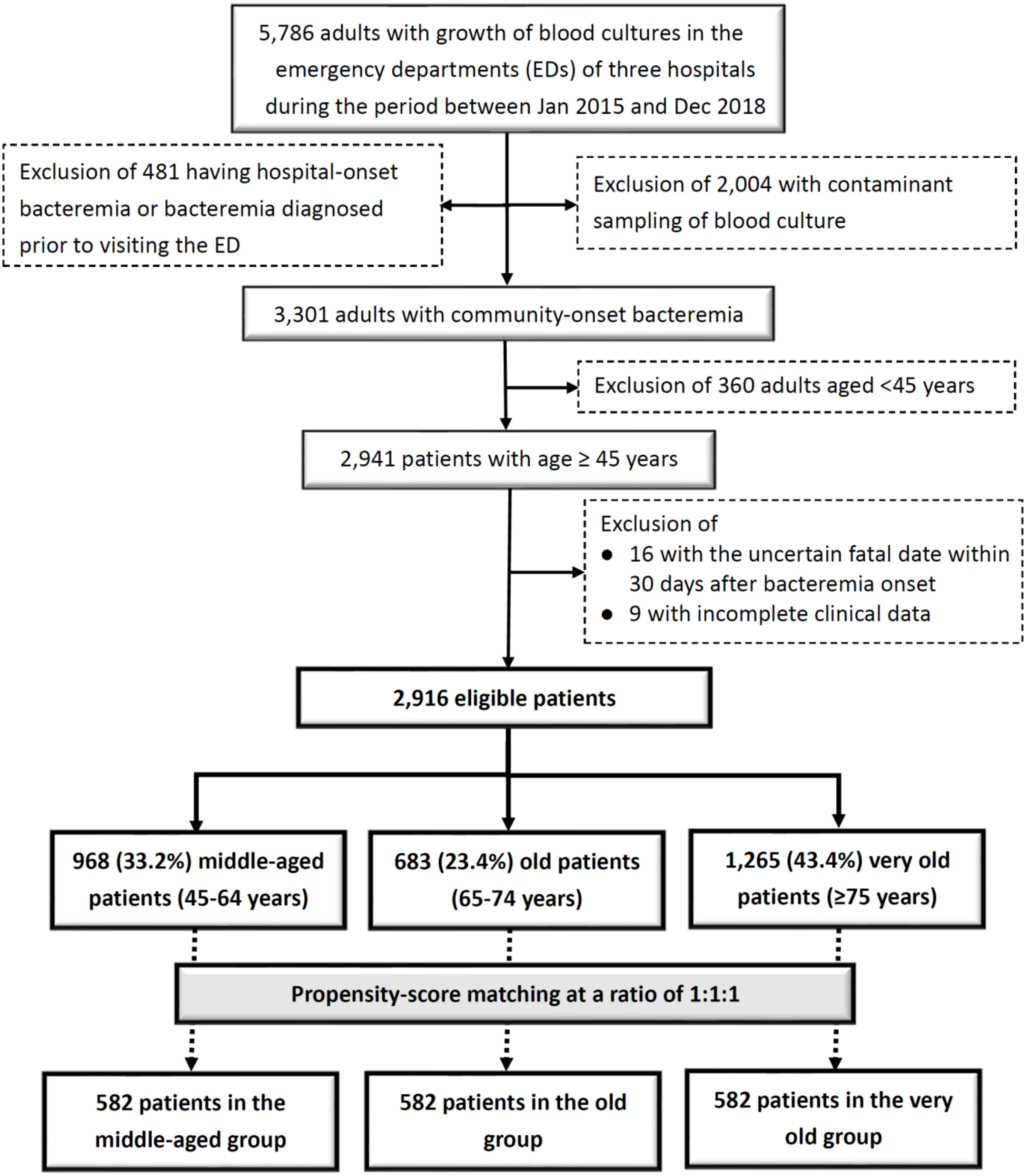

Supplemental Table 1. Major classes of empirical antibiotics in the middle-aged, old, and very old groups.

Clinical variable	Patient number (%)*			*P* value
	Middle-aged n = 968	Old n = 683	Very old n = 1265	
Major empirical antibiotic				
**Third-generation cephalosporin**	**368 (38.0)**	**283 (41.4)**	**448 (35.4)**	**0.03**
**First-generation cephalosporin**	**124 (12.8)**	**55 (8.1)**	**124 (9.8)**	**0.005**
Second-generation cephalosporin	123 (12.7)	87 (12.7)	172 (13.6)	0.79
Fourth-generation cephalosporin	107 (11.1)	70 (10.2)	126 (10.0)	0.70
**Aminopenicillin/BLI**	**87 (9.0)**	**54 (7.9)**	**146 (11.5)**	**0.02**
Fluoroquinolone	66 (6.8)	49 (7.2)	95 (7.5)	0.82
Glycopeptide	65 (6.7)	41 (6.0)	61 (4.8)	0.15
**Ureidopenicillin/BLI**	**29 (3.0)**	**31 (4.5)**	**71 (5.6)**	**0.01**
Carbapenem	27 (2.8)	24 (3.5)	42 (3.3)	0.67
Ureidopenicillin	21 (2.2)	20 (2.9)	31 (2.5)	0.62

BLI = β-lactamase inhibitor. Boldface indicates statistical significance ($P < 0.05$) in the univariable analysis.

Supplemental Table 2. Top 10 causative microorganisms in the middle-aged, old, and very old groups.

Microorganisms	Microorganism number (%)			*P* value
	Middle-aged n=1,065	Old n=762	Very old n=1,454	
Escherichia coli	**365 (34.3)**	**277 (36.4)**	**580 (39.9)**	**0.01**
Klebsiella pneumoniae	**167 (15.7)**	**124 (16.3)**	**164 (11.3)**	**0.001**
Staphylococcus aureus	**158 (14.8)**	**84 (11.0)**	**133 (9.1)**	**0.03**
Streptococcus agalactiae	30 (2.8)	17 (2.2)	30 (2.1)	0.45
Pseudomonas aeruginosa	27 (2.5)	27 (3.5)	54 (3.7)	0.24
Enterobacter cloacae	27 (2.5)	27 (3.5)	35 (2.4)	0.27
Proteus mirabilis	20 (1.9)	14 (1.8)	42 (2.9)	0.15
Salmonella enteritidis	20 (1.9)	9 (1.2)	19 (1.3)	0.38
Streptococcus pneumoniae	**14 (1.3)**	**12 (1.7)**	**39 (2.7)**	**0.04**
Enterococcus faecalis	**11 (1.0)**	**21 (2.8)**	**42 (2.9)**	**0.005**

Boldface indicates statistical significance ($P < 0.05$) in the univariable analysis.

Supplemental Table 3. Risk factors of 30-day crude mortality in overall patients.

Variable	Patient number (%)*		Univariable analysis		Multivariable analysis	
	Death n = 449	Survival n = 2467	OR (95% CI)	*P* value	Adjusted OR (95% CI)	*P* value
**Delayed AAT, hour, median (IQR)**	**2.5 (1.5 – 22.1)**	**2.0 (1.0 – 7.5)**	–	**<0.001**	**1.003 (1.002–1.004)**	**<0.001**
Gender, male	267 (59.5)	1220 (49.5)	1.50 (1.22–1.84)	<0.001	NS	NS
**Nursing-home residence**	**56 (12.5)**	**117 (4.7)**	**2.86 (2.05–4.01)**	**<0.001**	**1.71 (1.12 – 2.62)**	**0.01**
**Polymicrobial bacteremia**	**78 (17.4)**	**211 (8.6)**	**2.25 (1.70–2.98)**	**<0.001**	**1.56 (1.09 -2.24)**	**0.02**
**Pitt bacteremia score ≥ 4**	**290 (64.6)**	**319 (12.9)**	**12.28 (9.79–15.40)**	**<0.001**	**10.13 (7.82 – 13.12)**	**<0.001**
**Inadequate source control during antibiotic therapy**	**78 (17.4)**	**211 (8.6)**	**2.08 (1.30–3.33)**	**<0.001**	**2.87 (1.59 – 5.20)**	**<0.001**
Source of bacteremia						
**Pneumonia**	**173 (38.5)**	**274 (11.1)**	**5.02 (4.00–6.30)**	**<0.001**	**1.86 (1.37 – 2.51)**	**<0.001**
**Urinary tract infection**	**50 (11.1)**	**912 (37.0)**	**0.21 (0.16–0.29)**	**<0.001**	**0.42 (0.24 – 0.73)**	**0.002**
Biliary tract infection	22 (4.9)	240 (9.7)	0.48 (0.31–0.75)	0.001	NS	NS
**Liver abscess**	**7 (1.6)**	**91 (3.7)**	**0.41 (0.19–0.90)**	**0.02**	**0.35 (0.15 – 0.82)**	**0.02**
Causative microorganism						
Escherichia coli	112 (24.9)	1110 (45.0)	0.41 (0.32–0.51)	<0.001	NS	NS
Klebsiella pneumoniae	101 (22.5)	352 (14.3)	1.74 (1.36–2.24)	<0.001	NS	NS
Staphylococcus aureus	78 (17.4)	298 (12.1)	1.53 (1.17–2.01)	0.002	NS	NS
Pseudomonas aeruginosa	36 (8.0)	72 (2.9)	2.90 (1.92–4.38)	<0.001	1.66 (0.96 – 2.86)	0.07
**Fatal comorbidity (McCabe and Jackson classification)**	**214 (28.8)**	**235 (10.8)**	**3.33 (2.70 – 4.10)**	**<0.001**	**2.30 (1.70 – 3.12)**	**<0.001**
Comorbidity						
**Haemato-oncology**	**216 (48.1)**	**682 (27.6)**	**2.43 (1.98–2.98)**	**<0.001**	**1.70 (1.26 – 2.29)**	**<0.001**
Hypertension	214 (47.7)	1321 (53.5)	0.79 (0.65–0.97)	0.02	NS	NS
Diabetes mellitus	154 (34.3)	1014 (41.1)	0.71 (0.60–0.84)	0.007	NS	NS
Neurological disease	129 (28.7)	595 (24.1)	1.27 (1.01–1.59)	0.04	NS	NS
**Liver cirrhosis**	**84 (18.7)**	**281 (11.4)**	**1.79 (1.37–2.34)**	**<0.001**	**2.94 (2.04 – 4.24)**	**<0.001**

AAT = appropriate antimicrobial therapy; CI = confidence interval; IQR = interquartile range; OR = odds ratio; NS = not significant (by backward multivariable regression). Boldface indicates statistical significance ($P < 0.05$) in the logistic regression model.

*Data are expressed as numbers (%), unless indicated specifically.
